# Replicative study of GWAS reported TP63C/T rs710521, TERTC/T rs2736098 and SLC14A1C/T rs17674580 with susceptibility to bladder cancer in North Indians

**DOI:** 10.1186/1755-8166-7-S1-P16

**Published:** 2014-01-21

**Authors:** Vibha Singh, Praveen Kumar Jaiswal, Rakesh Kapoor, Rama Devi Mittal

**Affiliations:** 1 Department of Urology, Sanjay Gandhi Post Graduate Institute of Medical Science, Raebareli Road, Lucknow 226014, India

## Background

Genome Wide Association Studies (GWAS) have confirmed association of rs710521, rs2736098 and rs17674580 gene variants with susceptibility to Bladder Cancer (BC) in European and Caucasian. However, the risk conferred for BC in north Indians is unknown. In present study, we replicate GWAS findings of TP63C/T(rs710521), TERTC/T(rs2736098) and SLC14A1C/T(rs17674580) gene polymorphisms for their association with BC susceptibility in North Indian population.

## Material and methods

Three SNPs were genotyped by real-time polymerase chain reaction in histologically confirmed 225 BC cases and 240 healthy controls (age and gender matched) from North Indians in a hospital based study. To evaluate SNP effects on BC susceptibility, odds ratio and confidence interval were calculated by SPSSver.16.0. Bioinformatics analysis was done by F-SNP free online web server.

## Results

In case of TP63C/T(rs710521), variant genotype (TT) showed significant reduced risk for BC (p=0.045;OR=0.53). On Combining heterozygous and variant genotype, it showed reduced risk for BC (p**<**0.001;OR=0.54). In case of TERTC/T(rs2736098) heterozygous genotype (CT) as well as variant genotype (TT) showed significant risk for BC susceptibility (p=0.031;OR=1.77,p=0.004;OR=2.78 respectively) along with T allelic level (p<0.001;OR=4.19). Further SLC14A1C/T(rs17674580), variant genotype (TT) also showed significant high risk for BC susceptibility (p=0.006;OR=3.01) along with variant T allele level (p=0.003;OR=1.52). Interestingly smoking was also found to modulate risks for BC in case of TERT and SLC14A1 variant genotype (TT). Further tumor-grade-stage level of cases supports the genotypic data with TERT and SLC14A1 for BC risk. Bioinformatics analysis supported our result at genotypic level for the BC risk for TERTC/T and SLC14A1C/T.

## Conclusions

Our results indicated that TERTC/T(rs2736098) and SLC14A1C/T(rs17674580) showed high risk for BC in North Indian population. However, TP63C/T(rs710521) showed reduced risk of BC susceptibility. More study with large sample size and diverse ethnicity are required to validate our observations.

